# Thyroid nodule sizes influence the diagnostic performance of TIRADS and ultrasound patterns of 2015 ATA guidelines: a multicenter retrospective study

**DOI:** 10.1038/srep43183

**Published:** 2017-02-24

**Authors:** Ting Xu, Jing-yu Gu, Xin-hua Ye, Shu-hang Xu, Yang Wu, Xin-yu Shao, De-zhen Liu, Wei-ping Lu, Fei Hua, Bi-min Shi, Jun Liang, Lan Xu, Wei Tang, Chao Liu, Xiao-hong Wu

**Affiliations:** 1 Department of Endocrinology, the First Affiliated Hospital with Nanjing Medical University, Nanjing, China; 2 Department of Endocrinology, Jiangsu Province Official Hospital, Nanjing, China; 3 Department of Ultrasound, the First Affiliated Hospital with Nanjing Medical University, Nanjing, China; 4 Department of Endocrinology, Jiangsu Province Hospital on Integration of Chinese and Western Medicine, Nanjing, China; 5 Department of Endocrinology, Third Affiliated Hospital of Soochow University, Changzhou, China; 6 Department of Endocrinology, Affiliated Hospital of Soochow University, Suzhou, China; 7 Department of Endocrinology, Huai’an First People’s Hospital, Huai’an, China; 8 Department of Endocrinology, The Central Hospital of Xuzhou Affiliated to Xuzhou Medical College, Xuzhou, China; 9 Department of Endocrinology, WuXi People’s Hospital, Wuxi, China

## Abstract

To evaluate the impact of thyroid nodule sizes on the diagnostic performance of thyroid imaging reporting and data system (TIRADS) and ultrasound patterns of 2015 American Thyroid Association (ATA) guidelines. Total 734 patients with 962 thyroid nodules were recruited in this retrospective study. All nodules were divided into three groups according to the maximal diameter (d < 10 mm, d = 10–20 mm and d > 20 mm). The ultrasound images were categorized based on TIRADS and ATA ultrasound patterns, respectively. A total of 931 (96.8%) and 906 (94.2%) patterns met the criteria for TIRADS and ATA ultrasound patterns. The AUC (0.849) and sensitivity (85.3%) of TIRADS were highest in d = 10–20 mm group. However, ATA had highest AUC (0.839) and specificity (89.8%) in d > 20 mm group. ATA ultrasound patterns had higher specificity (*P* = 0.04), while TI-RADS had higher sensitivity (*P* = 0.02). In nodules d > 20 mm, the specificity of ATA patterns was higher than TIRADS (*P* = 0.003). Our results indicated that nodule sizes may influence the diagnostic performance of TIRADS and ATA ultrasound patterns. The ATA patterns may yield higher specificity than TIRADS, especially in nodules larger than 20 mm.

Thyroid nodules are common diseases, especially in regions with inadequate iodine supply. However, only 10–15% of these nodules are malignant^1^. How to distinguish these malignant lesions is a great clinical diagnostic challenge. High-resolution ultrasound (US) is recommended as the first line modality in the evaluation of thyroid nodules. Several US characteristics have been proposed to be associated with an increased risk of malignancy such as microcalcifications, hypoechogenecity, irregular margins, taller-than-wide shape and intranodular vascularity. However, none of them allows to reliably distinguish malignancy from benign nodules^2^. In 2009, Horvath established Thyroid Imaging Reporting and Data System (TIRADS) based on specific patterns composed of two or more features^3^. The model standardized and simplified the reports and unified codes between radiologists and endocrinologists and offered good diagnostic performance with the high sensitivity and PPV of 88% and 94%, respectively. In the same year, Park *et al*.^4^ proposed an equation for predicting the probability of malignancy in thyroid nodules based on 12 ultrasound features. In 2013, Kwak *et al*.^5^ published a new TIRADS classification based on the number of suspicious ultrasound features. As the number of suspicious US features increased, the fitted probability and risk of malignancy also increased. A recent meta-analysis of TIRADS showed that the sensitivity and specificity was 0.79 and 0.71, indicating that the TIRADS categories were a promising tool to evaluate thyroid benign and malignant nodules for making preoperative decision^6^.

Recently, the 2015 American Thyroid Association (ATA) guidelines constructed a new ultrasound risk stratification model from very low suspicion to high suspicion for malignancy according to sonographic features^7^. Yoon *et al*. have compared the diagnostic efficiency between the new ATA ultrasound patterns and the Korean TIRADS proposed in differentiating malignancy from benign lesions, indicating that ATA classifications may yield higher specificity, while TIRADS may offer a relatively higher sensitivity^8^. However, the influence of nodule sizes on the performance of these models has not been well investigated. The purpose of our study was to evaluate the diagnostic performance of original TIRADS developed by Horvath and ATA ultrasound patterns in thyroid nodules and to further clarify the impact of thyroid nodule sizes on the two models.

## Results

### Patient findings

**Figure 1:**
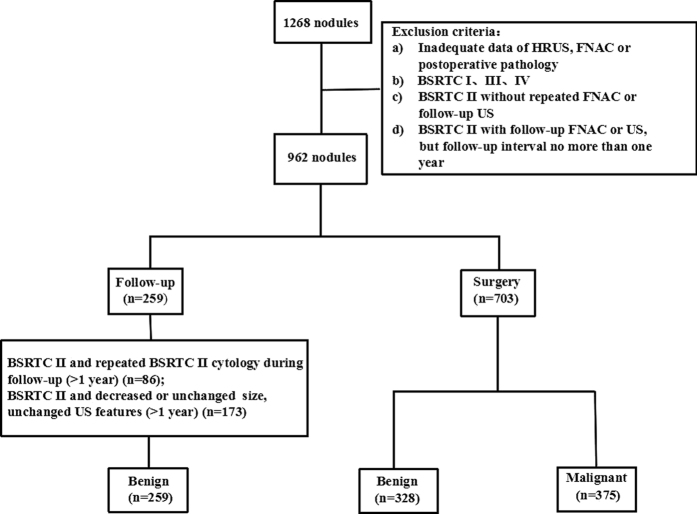
Diagram of the study group.

A total of 962 thyroid nodules in 734 patients were included in our study with 578 women and 156 men. The average age was 46.8 ± 13.1 years old and the mean diameter of the nodules was 17.7 ± 12.8 mm. All 375 malignant lesions and 328 benign nodules were confirmed by histopathology. The remaining 259 nodules were regarded as benign lesions due to the repeated benign cytology or follow-up ultrasound after the first benign cytology (Fig. 1). The epidemiological, clinical data of studied cases between three groups of different sizes were shown in Table 1. Malignancy rates, male gender, nodularity, FT3 level were significantly different in three groups. While location, lymphadenopathy, age, FT4 level, TSH level showed no statistical difference between the groups (*P* > 0.05). The malignancy rates of nodules d > 20 mm, d = 10–20 mm and d < 10 mm were 22.2%, 45.7% and 48.5%, respectively.

### Correlations between the TI-RADS classification and final diagnosis

A total of 931 patterns (96.8%) were able to be categorized based on TIRADS classification. The malignancy rates of TIRADS 2, 3, 4A, 4B and 5 were 0, 14.1% (62 of 439 nodules), 50.0% (118 of 236 nodules), 80.4% (156 of 194 nodules) and 100.0% (27 of 27 nodules), respectively, with significant differences between categories (P < 0.001). The correlations between the TIRADS classification and final diagnosis according to nodule size were shown in Table 2. The ROC curves demonstrated that the best cutoff of TI-RADS was IV in all three groups. The sensitivity, specificity and AUC in d < 10 mm group were 82.5%, 57.7% and 0.753, respectively. In d = 10–20 mm group, the sensitivity, specificity and AUC increased to 85.3%, 72.6% and 0.849. The sensitivity and AUC were the highest among the three groups. In d > 20 mm group, TIRADS had lowest sensitivity (76.9%), highest specificity (80.6%) and relatively higher AUC (0.836) (Table 3).

The remaining 31 nodules couldn’t be categorized, of which 12 (38.7%) nodules were validated as PTCs by surgery. Among them, there was 11, 12, 8 cases in d < 10 mm, 10–20 mm and > 20 mm group, respectively, indicating that nodule size had no influence on this aspect. Moreover, in these nodules beyond the range of TIRADS classifications, nodules with hypoechogenicity and tall-than-wide shape had 100% malignancy risk, hypoechogenicity accompanied with irregular shape and ill-defined margin were likely to have 75.0% malignancy rate.

### Correlations between ultrasound patterns of 2015 ATA guidelines and final diagnosis

A total of 906 patterns (94.2%) were able to be categorized based on ATA ultrasound patterns. According to histopathology or follow-up results, the malignancy rates of the nodules with very low, low, intermediate, and high suspicion for malignancy were 5.3%, 10.0%, 21.8% and 71.8%, respectively, with significant differences between patterns (*P* < .001). The correlations between the ATA ultrasound patterns and final diagnosis according to size were shown in Table 4. The ROC curves demonstrated that the best cutoff of ATA ultrasound patterns was High suspicious for malignancy in all three groups. The sensitivity, specificity and AUC in d < 10 mm group were 80.5%, 63.7% and 0.721, respectively. In d = 10–20 mm group, the specificity and AUC increased to 79.9% and 0.813 at the cost of a decreased sensitivity (75.8%). In d > 20 mm group, ATA ultrasound patterns had the highest specificity (89.8%), AUC (0.839) and the lowest sensitivity (70.8%).

In terms of the remaining 56 nodules beyond the range of ATA patterns, 16 (28.6%) nodules were validated as PTCs by surgery. There was 17, 20, 19 cases in d ≤ 10 mm, 10–20 mm and > 20 mm group, respectively, indicating that nodule size had little relations with the nodules that couldn’t be classified by ATA ultrasound patterns. Furthermore, we compared ultrasound features of these benign and malignant lesions and found that hyper-/isoechogenecity accompanied with irregular shape had much tendency to be malignant (42.9%).

### Comparison of TIRADS and ATA ultrasound patterns in diagnostic value

Compared to TIRADS, the 2015 ATA guidelines yielded a significant higher specificity (79.6% vs 71.5%, *P* = 0.04), while TIRADS had a higher sensitivity (83.2% vs 77.3%, *P* = 0.02). The AUC was higher in TIRADS than 2015 ATA classification, though, not significantly (0.826 vs 0.807, *P* > 0.05).

In d < 10 mm group, the differences in diagnostic value between TIRADS and ATA ultrasound patterns were not significant (AUC: 0.753 vs 0.721, *P* = .25, sensitivity: 82.5% vs 80.5%, *P* > 0.05, specificity: 57.7% vs 63.7%, *P* > 0.05). In d = 10–20 mm group, there were no significant difference between the two models (AUC: 0.849 vs 0.813, *P* > 0.05, sensitivity: 85.3% vs 75.8%, *P* > 0.05, specificity: 72.6% vs 79.9%, *P* > 0.05). While in d > 20 mm group, the specificity of ATA ultrasound patterns was significantly higher compared with TIRADS classification (89.8% vs 80.6%, *P* = 0.003), while the sensitivity and AUC showed no significant difference between the two models (76.9% vs 70.8%, *P* > 0.05, 0.836 vs 0.839, *P* > 0.05) (Table 3).

## Discussion

In this study, we evaluated the impact of thyroid nodule sizes on the diagnostic performance of newly published ultrasound patterns of 2015 ATA guidelines and original TIRADS classifications. We found that TIRADS performed best for differentiating nodules between 10–20 mm, while ATA ultrasound patterns had best value in lesions larger than 20 mm. The ATA ultrasound patterns may yield higher specificity, especially in nodules larger than 20 mm.

TIRADS established by Horvath had been widely applied in clinical setting for the evaluation of thyroid nodules. Based on 10 US patterns, TIRADS related the rate of malignancy according to the patterns^3^. The malignant rates of TIRADS 3, 4A in the present study were 14.1%, 50.0%, pretty higher than the recommended range (< 5%, 5–10%, respectively), but equal to Cheng’s results^9^. Meanwhile, the diagnostic sensitivity and NPV in our research were 83.2%, 86.7%, much lower than Cheng’s results, but comparable to those of Horvath’s study^3^,^9^. This may be due to the difference of radiologists’ experience, study population, inter-observer variability, US criteria and devices. A recent meta-analysis of TIRADS found that the sensitivity and specificity was 0.79 and 0.71, which was equal to our results^6^. However, there were 3.3% patterns of nodules didn’t meet the criteria of the original TIRADS classification in our study, including some patterns of partial cyst, which accounted for 15–53.8% of all sonographically detected nodules^10^, or patterns of hypoechogenecity accompanied with taller-than-wide shape. The malignancy rate of these nodules reached 38.7%, within the recommended range of TIRADS 4B. Lesions with hypoechogenicity, irregular shape and ill-defined margin or hypoechogenicity with taller-than-wide shape have much tendency to be malignant. Thus, closer follow-up or fine-needle aspiration for these nodules should be applied.

The 2015 ATA guidelines for patients with thyroid nodules established a 5-tier risk classification of ultrasound patterns by combining several individual sonographic characteristics^7^. The malignancy rates of benign, very low to high suspicion for thyroid cancer were < 1%, < 3%, 5–10%, 10–20% and >70–90%, respectively. In our study, the malignancy risks were 71.8% for the high suspicion pattern, 21.8% for the intermediate-suspicion pattern and 10.0% for the low-suspicion pattern, which was comparable with the range in the 2015 ATA guidelines. However, the remaining 56 nodules (5.8%) were unable to be categorized based on the ATA ultrasound patterns, most of which showed patterns of hyper-/isoechogenecity with at least one suspicious feature like irregular shape, ill-defined margin, microcalcification or taller-than-wide shape. Though, many studies found that hyperechogenecity was a predictor of benign lesions^11^,^12^, Seo *et al*. considered that solid iso/hyperechoic nodule with any calcification beard a malignancy risk of 24.7%^13^. Those patterns of iso, nonencapsulated nodules with multiple peripheral microcalcifications that were beyond the range of ATA could be classified as TIRADS 4B with the malignancy risks around 10–80%. Among the 56 nodules in our study, 16 (28.6%) were proved to be PTCs pathologically, indicating that high malignancy risk could still exit in iso/hyperechoic nodules when they accompanied with some high-risk ultrasound features such as irregular shape.

Recently, Yoon *et al*.^8^ had applied both the 2015 ATA ultrasound patterns and the Korean TIRADS established by Kawk to the 1293 thyroid nodules (d ≥ 10 mm). They found that the sensitivity was higher with TIRADS (*P* = 0.024), whereas specificity, PPV, and accuracy were higher with the ATA guidelines (*P* < .001 for all). Similar to Yoon’s study, our study found that original TIRADS model had a higher sensitivity (*P* = 0.02), while specificity were higher with the ATA ultrasound patterns (*P* = 0.04). In addition, Yoon *et al*. also revealed that 44 (3.4%) patterns did not meet the criteria for any ATA pattern including hyper- to isoechoic solid or partially cystic nodules with microlobulated or irregular margins, microcalcifiations or mixed calcifiations, or nonparallel shape and the malignancy risk was 18.2%.

The novel finding in our study was that nodular size markedly influenced the diagnostic performance of the TIRADS and 2015 ATA US patterns in differentiating benign and malignant lesions. The value of US between large lesions and small ones was controversial. Andrej *et al*.^14^ performed multivariate logistic regression analysis to evaluate the accuracy of US criteria for thyroid cancer in lesions d ≤ 15 mm and d > 15 mm, finding that the accuracy of US differentiation among larger nodules was lower than that among smaller ones. However, in Moon’s study^11^, the specificity and PPV of ultrasound in nodules larger than 10 mm were greatly higher than those in smaller nodules with a little decreased sensitivity. In our study, TIRADS in nodules d < 10 mm had a lowest AUC and specificity among the three groups, in line with the conclusions of Cheng *et al*.^9^ that TIRADS model of thyroid nodules was less reliable in smaller lesions. Interestingly, ATA ultrasound patterns also played a less credible role in nodules d < 10 mm. However, the difference was that the TIRADS performed best in nodules d = 10–20 mm, while the AUC and specificity of ATA patterns were highest in lesions d > 20 mm. The diagnostic value between the two models was similar in smaller size subgroups including d < 10 mm and 10–20 mm. Nevertheless, in nodules larger than 20 mm, the sensitivity of TIRADS was higher than ATA ultrasound patterns, though not significant, while the specificity of ATA patterns was significantly superior to TIRADS.

The limitations of our study should also be addressed. Firstly, all classifications were performed based on the static images of US, which might cause misinterpretation of ultrasound classification. Secondly, description of features was reported by different radiologists, which may cause inter-observer variability. Thirdly, this was a retrospective study, the selection bias such as patients who underwent thyroid surgery and gender bias (female: male = 3.71) may cause the high percentage of carcinomas (39.0%), resulting in the overestimation of PPV and underestimation of NPV, both in TIRADS and ATA ultrasound patterns^15^. However, this was a general limitation of most studies performed at endocrinology centers^16^,^17^. Fourthly, 259 of the 962 nodules (26.9%) were regarded as benign lesions based on cytology and follow-up US, which may cause false negative results.

In conclusion, both TIRADS and the 2015 ATA ultrasound patterns provide effective malignancy risk stratification for thyroid nodules. Nodule sizes may influence the diagnostic performance of the two models. The TIRADS showed best value in nodule between 10–20 mm, while ATA patterns had highest value in lesions larger than 20 mm. Both models are less reliable in lesions smaller than 10 mm. The ATA patterns may yield higher specificity than TIRADS, especially in nodules larger than 20 mm. Those nodules beyond the range of TIRADS categories and ATA patterns had little relation with nodule size and may still have a relatively high risk of malignancy (38.7% and 28.6%). However, due to the limitations of this study, our findings still need to be further validated in the clinical practice.

## Methods

### Subjects

This retrospective study was based on patient data collected from eight tertiary hospitals around Jiangsu province in China from January 6, 2014 to December 20, 2014. A total of consecutive patients underwent US-guided FNAB or thyroidectomy for thyroid nodules. Patients who met the following criteria by reviewing US patterns and clinical data were included in this study: (*a*) patients who underwent thyroid surgery regardless of cytologic results, (*b*) patients who underwent fine-needle aspiration cytology at least two times within a 1-year interval for benign thyroid lesions, (*c*) patients who had benign results on cytology and showed no change or decreased size at follow-up US for at least a year (7). The increase in size was defined as more than a 50% change in volume or a 20% increase in at least two nodule dimensions with a minimal increase of 2 mm in solid nodules or in the solid portion of mixed cystic-solid nodules (8). A total of 734 patients with 962 nodules (mean age, 46.75 ± 14.09 years; range, 15–84 years) were included preliminarily. There were 156 men (mean age, 50.41 ± 13.60 years; age range, 17–73 years) and 578 women (mean age, 45.76 ± 12.18 years; age range, 15–84 years). All nodules were divided into three groups according to the maximal diameter (d < 10 mm, d = 10–20 mm and d > 20 mm). Informed consent was obtained from all patients and the study was performed in accordance with the ethical guidelines of the Helsinki Declaration and approved by the First Affiliated Hospital with Nanjing Medical University ethics review committee (2012-SR-058).

### US examination technique

All US images were obtained by using a 4–13 MHz linear array transducer. The scanning protocol in all cases included both transverse and longitudinal real-time imaging of the thyroid nodules. Participants were asked to assess the thyroid nodules according to the criteria from published literature^18^,^19^,^20^. The features used in analysis included size, composition, echogenicity of solid portion, orientation, shape, margin, and calcifications. All static US patterns and description of features were available and analyzed by a radiologist with 10 years of experience in thyroid imaging. Clinical information and pathology results were not available to the radiologist.

#### TIRADS classification

As the original TIRADS proposed by Horvath was widely used in our center, so all nodules were categorized according to TIRADS classification as follows^3^. TIRADS 2: Anechoic with hyperechoic spots, nonvascularized lesion. Nonencapsulated, mixed, nonexpansile, with hyperechoic spots, vascularized lesion, grid aspect (spongiform nodule). Nonencapsulated, mixed with solid portion, isoechogenic, expansile, vascularized nodule with hyperechoic spots. TIRADS 3: Hyper, iso, or hypoechoic, partially encapsulated nodule with peripheral vascularization, in Hashimoto’s thyroiditis. TIRADS 4A: Solid or mixed hyper, iso, or hypoechoic nodule, with a thin capsule. Hypoechoic lesion with ill-defined borders, without calcifications. Hyper, iso, or hypoechoic, hypervascularized, encapsulated nodule with a thick capsule, containing calcifications (coarse or microcalcifications). TIRADS 4B: Hypoechoic, nonencapsulated nodule, with irregular shape and margins, penetrating vessels, with or without calcifications. TIRADS 5: Iso or hypoechoic, nonencapsulated nodule with multiple peripheral microcalcifications and hypervascularization.

#### Ultrasound patterns of 2015 ATA guidelines

All nodules were scored based on ultrasound patterns of 2015 ATA guidelines as follows^7^: Benign: Purely cystic nodules. Very Low Suspicion: Spongiform or partially cystic nodules without any of the sonographic features described in low, intermediate or high suspicion patterns. Low Suspicion: Isoechoic or hyperechoic solid nodule, or partially cystic nodule with eccentric solid areas, without microcalcification, irregular margin or extrathyroidal extension, or taller than wide shape. Intermediate Suspicion: Hypoechoic solid nodule with smooth margins without microcalcifications, extrathyroidal extension, or taller than wide shape. High Suspicion: Solid hypoechoic nodule or solid hypoechoic component of a partially cystic nodule with one or more of the following features including irregular margins (infiltrative, microlobulated), microcalcifications, taller than wide shape, rim calcifications with small extrusive soft tissue component, evidence of extrathyroidal extension.

### Statistical analysis

Statistical analysis was performed using SPSS 20.0 software (SPSS Inc., Chicago, USA). All quantitative values were expressed as means ± SD. Differences in the values of continuous variables between three groups were evaluated by the one-way ANOVA test or non-parametric test. Differences in the distribution of categorical variables between groups were evaluated by the 2-tailed Chi-square (χ^2^) test or Fisher exact test. Compared to the final diagnosis (according to pathology or follow-up results), the sensitivity, specificity, positive predictive value (PPV) and negative predictive value (NPV) were calculated for each method. Receiver Operating Characteristic (ROC) curve analysis with MedCalc 11.4.2.0 software (MedCalc Software, Ostend, Belgium) was used to compare the two models and to determine the optimal cut-off value between benign and malignant nodules. Area under the curves (AUCs) and *P* value were calculated. *P* < 0.05 was considered significant in all tests.

## Additional Information

**How to cite this article**: Xu, T. *et al*. Thyroid nodule sizes influence the diagnostic performance of TIRADS and ultrasound patterns of 2015 ATA guidelines: a multicenter retrospective study. *Sci. Rep.* **7**, 43183; doi: 10.1038/srep43183 (2017).

**Publisher's note:** Springer Nature remains neutral with regard to jurisdictional claims in published maps and institutional affiliations.

## Figures and Tables

**Table 1:** Clinical features of the study population and basic characters of the nodules.

Features		<10 mm	10–20 mm	>20 mm	*P* value
Pathology	Benign	184	157	246	0.000
	Malignant	173	132	70	
Sex	Male	46	54	56	0.000
	Female	181	183	214	
Nodularity	Single	140	147	180	0.000
	Multiple	217	142	136	
Lymphadenopathy	Present	277	219	254	0.422
	Absent	80	70	62	
Location	Isthmus	15	12	6	0.189
	Left	174	126	161	
	Right	168	151	149	
Age (year)		45.7 ± 12. 5	46.4 ± 13.6	48.2 ± 13.2	0.147
FT3 (pmol/L)		5.2 ± 3.7	4.8 ± 1.6	5.0 ± 3.2	0.010
FT4 (pmol/L)		16.6 ± 11.3	15.5 ± 5.6	14.9 ± 3.8	0.173
TSH (mIU/L)		2.2 ± 1.8	2.8 ± 2.8	2.8 ± 6.9	0.087

**Table 2:** The malignancy rates of TI-RADS classifications.

	n	Surgery (%)	Follow-up (%)	Benign (%)	Malignant (%)	*P* value
**d < 10 mm**						0.000
2	4	2 (50.0)	2 (50.0)	4 (100.0)	0	
3	131	91 (69.5)	40 (30.5)	103 (78.6)	28 (21.4)	
4A	105	83 (79.0)	22 (21.0)	48 (45.7)	57 (54.3)	
4B	98	77 (78.6)	21 (21.4)	22 (22.4)	76 (77.6)	
5	8	8 (100.0)	0	0	8 (100.0)	
Total	346	261 (75.4)	85 (24.6)	177 (51.2)	169 (48.8)	
**10–20 mm**						0.000
2	2	1 (50.0)	1 (50.0)	2 (100.0)	0	
3	125	97 (77.6)	28 (22.4)	107 (85.6)	18 (14.4)	
4A	80	69 (86.3)	11 (13.7)	35 (43.8)	45 (56.2)	
4B	56	56 (100.0)	0	5 (8.9)	51 (91.1)	
5	14	14 (100.0)	0	0	14 (100.0)	
Total	277	237 (85.6)	40 (14.4)	149 (53.8)	128 (46.2)	
**d > 20 mm**						0.000
2	29	10 (34.5)	19 (65.5)	29 (100.0)	0	
3	183	107 (59.3)	76 (40.7)	167 (91.5)	16 (8.5)	
4A	51	26 (51.0)	25 (49.0)	35 (68.6)	16 (31.4)	
4B	40	37 (92.5)	3 (7.5)	11 (27.5)	29 (72.5)	
5	5	5 (100.0)	0	0	5 (100.0)	
Total	308	185 (60.1)	123 (39.9)	242 (78.6)	66 (21.4)	

**Table 3:** The comparison of TI-RADS classifications and 2015 ATA guidelines in diagnostic value.

	Cut-off	AUC (95% CI)	Sensitivity (95% CI)	Specificity (95% CI)	PPV (95% CI)	NPV (95% CI)
**d < 10 mm**						
TI-RADS classification	4A	0.753 (0.716–0.805)	82.5 (76.3–88.7)	57.7 (49.8–65.6)	66.4 (59.1–72.7)	77.3 (68.7–84.5)
2015 ATA guidelines	High suspicious for malignancy	0.721 (0.670–0.772)	80.5 (73.2–86.2)	63.7 (55.5–71.2)	69.2 (62.0–75.8)	76.0 (67.6–83.1)
**10–20 mm**						
TI-RADS classification	4A	0.849 (0.802–0.891)	85.3 (78.4–91.3)	72.6 (64.7–79.8)	73.5 (65.5–80.4)	85.4 (77.7–91.0)
2015 ATA guidelines	High suspicious for malignancy	0.813 (0.749–0.849)	75.8 (66.0–82.9)	79.9 (72.0–86.1)	76.5 (68.3–84.0)	77.6 (70.8–85.1)
**d > 20 mm**						
TI-RADS classification	4A	0.836 (0.790–0.876)	76.9 (64.8–86.5)	80.6 (75.0–85.4)	52.1 (41.6–62.4)	92.7 (88.3–95.9)
2015 ATA guidelines	High suspicious for malignancy	0.839 (0.792–0.879)	70.8 (58.2–81.4)	89.8 (85.1–93.4)	66.7 (54.3–77.6)	91.4 (86.9–94.8)

**Table 4:** The malignancy rates of 2015 ATA guidelines ultrasound patterns.

	n	Surgery (%)	Follow-up (%)	Benign (%)	Malignant (%)	*P* value
**d < 10 mm**						0.000
Very low suspicion	5	4 (80.0)	1 (20.0)	4 (80.0)	1 (20.0)	
Low suspicion	35	23 (65.7)	12 (34.3)	29 (82.9)	6 (17.1)	
Intermediate suspicion	107	78 (72.9)	29 (27.1)	82 (76.6)	25 (23.4)	
High suspicion	193	155 (80.3)	38 (19.7)	57 (29.5)	136 (70.5)	
Total	340	260 (76.5)	80 (23.5)	172 (50.6)	168 (49.4)	
**10**–**20 mm**						0.000
Very low suspicion	9	4 (44.4)	5 (55.6)	8 (88.9)	1 (11.1)	
Low suspicion	60	35 (58.3)	25 (41.7)	53 (88.3)	7 (11.7)	
Intermediate suspicion	76	50 (65.8)	26 (34.2)	54 (71.1)	22 (28.9)	
High suspicion	124	100 (80.6)	24 (19.4)	29 (23.4)	95 (76.6)	
Total	269	189 (70.3)	80 (29.7)	144 (53.5)	125 (46.5)	
**d > 20 mm**						0.000
Very low suspicion	24	16 (66.7)	8 (33.3)	24 (100.0)	0 (0)	
Low suspicion	116	78 (67.2)	38 (32.8)	108 (93.1)	8 (6.9)	
Intermediate suspicion	88	58 (65.9)	30 (34.1)	76 (86.4)	12 (13.6)	
High suspicion	69	60 (87.0)	9 (13.0)	23 (33.3)	46 (66.7)	
Total	297	212 (71.4)	85 (28.6)	231 (77.8)	66 (22.2)	
